# Assessment of the Quality of Life of Saudi Patients in Pharmacist-Led Heart Failure Clinics: A Cross-Sectional Observational Study

**DOI:** 10.7759/cureus.92887

**Published:** 2025-09-21

**Authors:** Aseel A Alsryea, Ali M Alhomoud, Abdu J Abbadi, Ahmed O Ajibi, Amjad A Judayba, Noura Y Harbi, Amal A Almutairi, Khalid A Ageeli, Raneem A Alodhaib, Ziyad E Hakami, Ali T Alasmari, Ibrahim Y Alghubayri

**Affiliations:** 1 Pharmaceutical Care Administration, Armed Forces Hospital Southern Region, Khamis Mushayt, SAU; 2 Pharmacy Service Department, King Faisal Hospital and Research Center, Jeddah, SAU; 3 Pharmaceutical Care Administration, Najran Armed Forces Hospital, Najran, SAU; 4 Pharmaceutical Care Administration, Najran Armed Forces Hospital, Khamis Mushayt, SAU; 5 Nursing Administration, Prince Sultan Cardiac Center, Riyadh, SAU; 6 College of Pharmacy, Jazan University, Jazan, SAU; 7 Pharmacy Department, King Fahad Specialist Hospital, Dammam, SAU

**Keywords:** cross-sectional, heart failure, hf, qol, quality of life

## Abstract

Objectives

Heart failure (HF) is a global health challenge with high morbidity and mortality, significantly impacting patients' quality of life (QOL). The QOL of Saudi patients, who were treated in the pharmacist-led HF clinics, is unknown. This study aims to evaluate HF patients' QOL using the validated Kansas City Cardiomyopathy Questionnaire (KCCQ-12).

Methods

A cross-sectional observational study was conducted with 111 randomly selected HF patients. QOL was assessed using the KCCQ-12, which encompasses domains evaluating physical limitations, HF-specific symptoms (swelling, shortness of breath (SOB), and fatigue), QOL, and social impact, with a score ranging from 0 (poorest health) to 100 (best health). The interpretation of health status is based on the overall summary KCCQ score as follows: very poor to poor (0-24), poor to fair (25-49), fair to good (50-74), and good to excellent (75-100).

Results

Overall, the highest score was in symptom frequency (82.7), indicating a few symptoms. The score for the dimension Social Limitation was 79.4, reflecting minimal social restrictions, while that for Physical Limitation was 71.7, indicating moderate physical activity limitations. The reported QOL score was 68.9, reflecting their fair to good QOL. The summary score was 75.4, reflecting an overall positive QOL assessment. The distribution of pharmacist interventions included the following: patient counseling was performed in 111 (100%) of cases, while the guideline-directed medical therapy (GDMT) monitoring follow-up was done for 58 (52.25%) of patients, and the initiation and titration of GDMT were conducted in 52 (46.85%) and 41 (36.94%) of cases, respectively. There is a weak positive correlation (rho=0.183) between the KCCQ-12 Summary Score and the total number of interventions received by patients, with a p-value of 0.056. This suggests a trend where an increased number of interventions may be associated with a slightly higher QOL, although this correlation is not statistically significant (p>0.05).

Conclusion

Based on the KCCQ findings, this study demonstrates the significant influence of clinical pharmacist interventions on the HF patient's QOL, which identifies the importance of HF clinics run by a cardiology clinical pharmacist within a multidisciplinary team.

## Introduction

Heart failure (HF) presents a significant global health challenge, characterized by its chronic and progressive nature, leading to substantial morbidity and mortality, alongside a detrimental impact on the patients' quality of life (QOL). According to a 2013 report from the Ministry of Health of Saudi Arabia, approximately 42% of the deaths in the country are attributed to cardiovascular disease (CVD) [1].

The National Health and Nutrition Examination Survey (NHANES) data also reveal that between 2013 and 2016, an estimated 6.2 million American adults aged ≥20 years (2.2%) were diagnosed with HF, compared to 5.7 million between 2009 and 2012, with approximately 400,000 new cases reported annually [2,3]. In Saudi Arabia, it's estimated that there are 455,222 cases of HF, with an annual incidence of 32,200 cases. A recent study indicated that 20% of patients admitted to Saudi hospitals with acute coronary syndrome (ACS) also had HF [4].

HF contributes significantly to mortality, with over 280,000 deaths attributed to it in 2008 [5]. The American Heart Association reports that about 42.3% of individuals diagnosed with HF die within five years of diagnosis [6,7]. The economic burden is substantial, with an estimated one million hospitalizations annually and costs totaling $32 billion per year in 2010 [8].

HF readmission rates are alarmingly high, with figures as high as 44% within the first six months and more than two-thirds within the first 12 months. Efforts to reduce readmission are crucial [9,10]. A retrospective cohort study in Saudi Arabia found readmission rates of 39.6% for 30 days and 37.6% for 90 days, with predictors including body mass index, hypertension, chronic kidney disease, hypothyroidism, dyslipidemia, and pulmonary disease [11].

Non-adherence to either medications or lifestyle modifications is a major cause of readmission and has been found to contribute to hospital admission in up to one-third of patients [9]. The rates of medication non-adherence in HF patients have varied widely, with most rates between 40% and 60% [12], and have also been associated with an increase in cardiac-related events, including emergency department (ED) visits, hospitalization, health care costs, and a reduction in QOL.

With their specialized knowledge in drug therapy, pharmacists are uniquely positioned to deliver essential pharmacotherapeutic interventions and recommendations for multidisciplinary teams. They can optimize medication regimens and lifestyle adjustments to enhance medication adherence, improve patient QOL, and ultimately, bolster therapeutic outcomes for individuals with HF [13].

Many studies have supported the positive and effective role of the pharmacist as a member of the multidisciplinary HF management team. These studies confirmed that the effective pharmacist contribution to the multidisciplinary HF team increases medication adherence [14], decreases the number of clinically relevant drug-drug interactions [15], and significantly reduces the risk of discrepancies and medication errors in the pharmacy discharge service [16,17], with the rate of adverse effects and other medication errors reduced by 25%-40%. The clinical pharmacist in the intensive care unit can reduce it by >60% [18-20], with patients achieving target doses of guideline-directed medical therapy (GDMT) and experiencing an improvement HF symptoms [21], thus leading to a reduction in the all-cause mortality and HF clinical events [22], improved patient knowledge about HF disease and patient QOL [23], reduced 30-day readmission rate through post-discharge or outpatient HF clinic counseling and regular follow-up.

Specialized HF clinics, facilitated by clinical pharmacists within multidisciplinary management teams alongside physicians and nurses, have emerged as pivotal endeavors in improving clinical outcomes for HF patients. These clinics offer extensive pharmacotherapeutic interventions such as medication management, patient education, monitoring and follow-up, implementation of evidence-based guideline recommendations, research, and quality improvement activities, in addition to collaboration with healthcare teams to ensure a comprehensive approach to HF management.

Despite their importance, there has been a notable absence of studies identifying the QOL for those patients who were treated in the HF clinic led by a cardiology clinical pharmacist as a part of a multidisciplinary team. This paper seeks to identify the QOL of the Saudi population in pharmacist-led HF clinics using the Kansas City Cardiomyopathy Questionnaire (KCCQ) as a primary objective of the study, with the knowledge that the most important pharmacotherapeutic interventions provided by cardiology clinical pharmacists to the HF multidisciplinary team are predicted to have an impact on the reported QOL as a secondary objective.

The ultimate aim of these initiatives is to bolster the effectiveness of HF clinics under the supervision of clinical pharmacists within multidisciplinary teams spanning various medical institutions. This effort seeks to expand the reach of these clinics, with the primary objectives being the reduction of HF readmission rates within the Saudi population and the enhancement of their QOL alongside a decrease in healthcare costs. Notably, the reported QOL among the Saudi population with HF is deficient across all domains of physical and mental components, with the reporting of left ventricular ejection fraction (LVEF) as the strongest predictor for the compromise in QOL [4].

## Materials and methods

A cross-sectional observational study design was used. The sample size was calculated using Epi Info software (Centers for Disease Control and Prevention (CDC), Atlanta, GA, USA) based on the expected mean total score of the KCCQ-12, estimated at 76.8 with a standard deviation of 22.3. To achieve a 95% confidence level with a margin of error of 5% (equivalent to 3.84 points), it was determined that a minimum of 130 participants would be required to provide adequate precision to estimate the mean KCCQ-12 score within the desired confidence interval. With a dropout rate of 14.6% not responding to the questionnaire, the recruited participants were 111 patients. The inclusion criteria were patients 18 years and older, a confirmed HF diagnosis by a cardiologist, treated in a pharmacist-led HF clinic, able to read and write in Arabic, and having the willingness to participate. Patients were excluded from the study if they had cognitive impairment, which made communication difficult and affected the ability to complete the questionnaire, or a terminal illness could affect their QOL regardless of HF. The sample was recruited from the largest specialized cardiac center in the middle province of Saudi Arabia, which receives referrals from all Saudi Arabia's cities and provides high-quality cardiac services to the Saudi population.

QOL was measured using the KCCQ. This questionnaire emerged as the most specific and commonly utilized self-reported questionnaire for evaluating the QOL in patients with congestive heart failure (CHF) [24]. A condensed version of the original KCCQ, consisting of 12 items, has been developed for clinical utility [25,26]. The KCCQ-12 encompasses domains evaluating physical limitations, HF-specific symptoms (including swelling, shortness of breath, and fatigue), quality of life, and the social ramifications of the disease [26]. These domains are the basis for generating two summary measures: the KCCQ-12 clinical summary score, which reflects patients' physical function and symptoms, and the KCCQ-12 overall summary score, encompassing physical, symptom, quality of life, and social function domains. The responses for each item are arranged sequentially, starting from 1 for the worst status and increasing numerically to represent better statuses (e.g., 2 for slightly better, 3 for even better, and so on). Each domain's score ranges from 0 to 100, where 0 signifies the poorest health status and 100 denotes the best. The interpretation of health status is based on the overall summary score of the KCCQ as follows: very poor to poor (0-24), poor to fair (25-49), fair to good (50-74), and good to excellent (75-100). The KCCQ has been extensively used in recent clinical trials to evaluate the health status of CHF patients and assess the impacts of different pharmacological therapies and innovative medical devices on their well-being. The KCCQ-12 questionnaire has been translated into Arabic and validated for Arabic-speaking HF patients in Saudi Arabia [27]. The permission to use this questionnaire was obtained from Dr. Ali Albarrati, the professor who validated the Arabic version.

The institutional review board (IRB) approval was obtained from the Prince Sultan Medical Military Research Center IRB Committee. HF patients who met the inclusion criteria and were treated in the pharmacist-led HF clinic were informed during their visit to the outpatient clinic about the study, and their willingness to answer the self-reported KCCQ-12 questionnaire was ascertained and their informed consent obtained. The participants agreed to answer the questionnaire using the online link, including the questionnaire and provide their contact number to answer their inquiries. Two trained nurse research assistants extracted patients’ demographic characteristics and medical variable data from patients’ medical records.

Data were analyzed using the Statistical Package for the Social Sciences for Windows 21.0 (IBM Corp, Armonk, NY). An alpha of <0.05 was used to determine the statistical significance of the analyses. Descriptive statistics (mean, standard deviation or frequency, and range) were used to assess participants' demographic and medical characteristics, as well as the KCCQ-12 scores.

Statistical analysis was conducted to examine the relationship between various predictors and QOL as measured by the KCCQ-12 summary score. Descriptive statistics, including means, standard deviations, and frequencies, were calculated to summarize the demographic and clinical characteristics of the participants. Normality tests were performed to assess the distribution of continuous data. For normally distributed data, parametric tests were utilized, while non-parametric tests were employed for data that did not meet normality assumptions. Specifically, the chi-square test was applied to evaluate associations between categorical variables, and the Mann-Whitney U test was used to compare differences in continuous variables between two groups. Furthermore, linear regression analysis was conducted to identify significant predictors of the KCCQ-12 summary score, allowing for a comprehensive understanding of the factors influencing QOL in patients with HF.

## Results

Characteristics of the HF patients

Table 1 presents the distribution of participants based on their social characteristics and comorbidities. The majority were married (n=98, 88.2%), with smaller proportions being single (n=10, 9.1%) or divorced (n=3, 2.7%). Age distribution showed that most participants were between 40 and 60 years (n=62, 55.5%), with 33 (30%) over 60 and 16 (14.5%) under 40. As regards educational level, the majority had a university education (n=62, 55.5%), followed by high school (n=27, 24.5%), elementary school (n=8, 18.2%), and middle school (n=10, 9.1%), with only four (3.6%) having higher education beyond university. Regarding comorbidities, 66 (60%) patients had diabetes mellitus (DM), while hypertension (HTN) affected 69 (62.7%) participants, and dyslipidemia (DLP) was present in 46 (41.8%) participants. Thyroid disorders were less common, involving eight (7.3%) patients. When smoking habit was assessed, 60 (53.6%) were found to be non-smokers, with 33 (30%) being current smokers and 18 (16.4%) former smokers. Most participants (n=82, 73.6%) had no readmission history, while 29 (26.4%) had at least one. Regarding the pharmacist intervention frequency per patient, 52 (47.3%) patients had three or more interventions, with three (n=30, 27.3%) being the most common. Finally, the KCCQ scores indicated that 66 (60%) participants reported a "Good to Excellent" quality of life, while 28 (24.5%) reported "Fair to Good," and 17 (15.5%) reported "Poor to Fair."

**Table 1:** Distribution of the participants according to their social characteristics and comorbidities DM: Diabetes mellitus, HTN: hypertension, DLP: dyslipidemia, LVEF: left ventricular ejection fraction, KCCQ: Kansas City Cardiomyopathy Questionnaire.

Variable	Subcategory	N	%
Marital Status	Divorced	3	2.7
	Married	97	88.2
	Single	10	9.1
Age Categories	<40	16	14.5
	40-60	61	55.5
	>60	33	30.0
Educational level	Elementary School Level	8	18.2
	High School Level	27	24.5
	Higher Education Level	4	3.6
	Middle School Level	10	9.1
	University Level	62	55.5
DM		66	60
LVEF%	≤40%	110	100
DLP		46	41.8
HTN		69	62.7
Thyroid disorders		8	7.3
Smoking	Non-smoker	60	53.6
	Current smoker	33	30.0
	Former smoker	18	16.4
History of Readmission	Yes	29	26.4
Number of Intervention/patients	No intervention	8	7.3
	One	5	4.5
	Two	28	25.5
	Three	30	27.3
	Four	23	20.9
	Five	13	11.8
	Six	2	1.8
	Seven	1	0.9
KCCQ summary score	Poor to Fair	17	15.5
	Fair to Good	27	24.5
	Good to Excellent	66	60.0

Distribution of the participants according to their KCCQ-12 score

**Figure 1:**
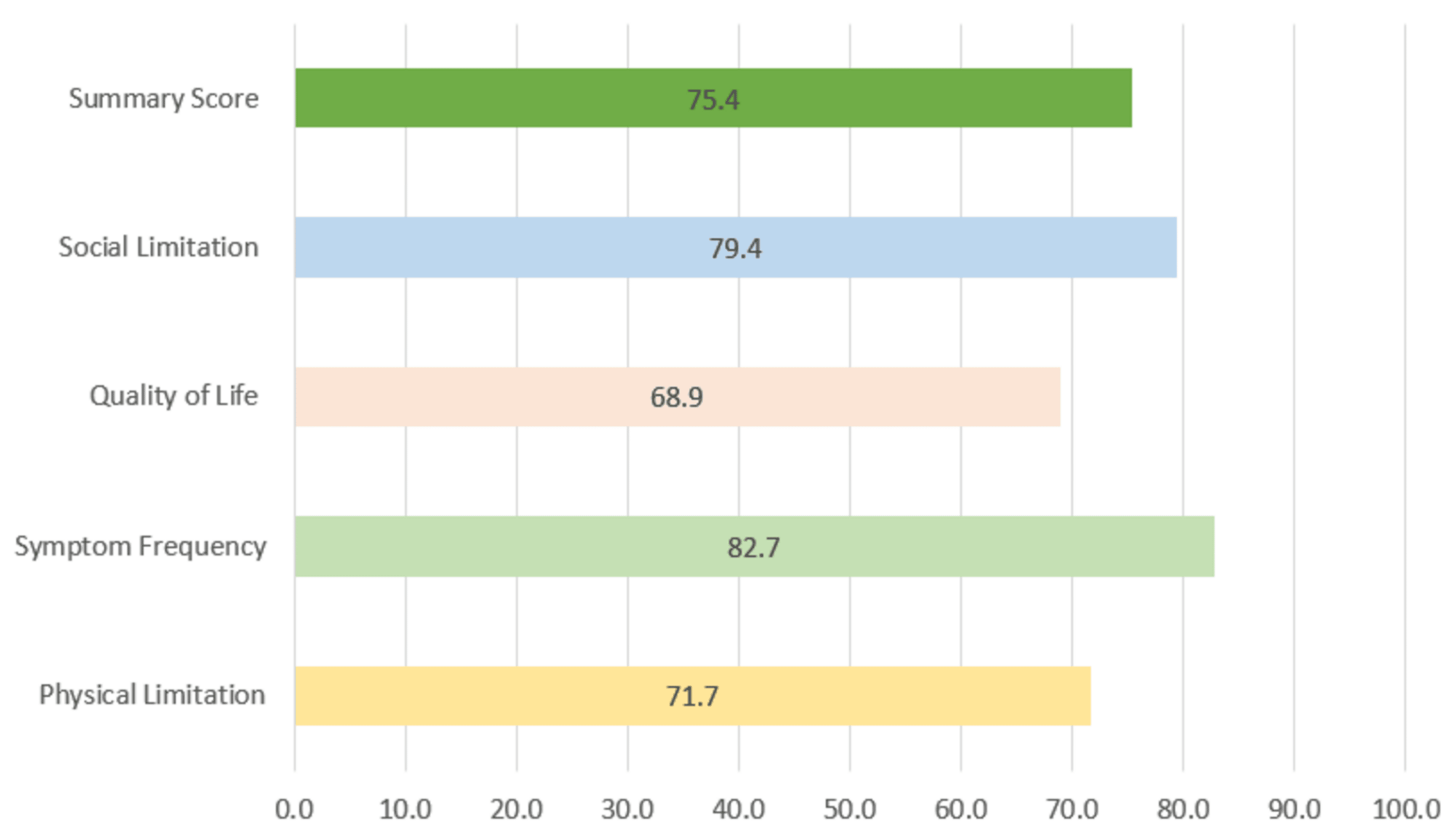
KCCQ-12 Domains: Total score KCCQ: Kansas City Cardiomyopathy Questionnaire

The KCCQ Score chart illustrates participants' health-related QOL across different domains. Overall, the highest score was in symptom frequency (82.7), indicating that participants experienced relatively few symptoms related to their condition. Social Limitation followed with a score of (79.4), suggesting that participants felt minimal social restrictions due to their health. Physical Limitation was moderately high at (71.7), suggesting that the patient experienced moderate limitations in their physical activities despite the ability to perform basic daily activities. The reported QOL score was 68.9, showcasing a fair to good QOL. The summary score was 75.4, reflecting an overall positive QOL assessment (Figure 1).

Correlation among KCCQ-12 domain scores and its summary score

The correlation analysis among the KCCQ-12 domain scores and the summary score reveals strong positive relationships across all domains. Specifically, the KCCQ-12 Physical Limitation domain shows significant positive correlations with the other domains: Symptom Frequency (SF), QOL, Social Limitation (SL), and the overall KCCQ-12 summary score (r=0.758, p<0.001). Similarly, the SF domain is strongly correlated with QOL (r=0.538, p<0.001), SL (r=0.757, p<0.001), and the summary score (r=0.757, p<0.001). The QOL domain also shows robust positive correlations with SL (r=0.765, p<0.001) and the summary score (r=0.811, p<0.001). The SL domain has the strongest correlation with the KCCQ-12 summary score, with a very high correlation (r=0.811, p<0.001). These results indicate that the KCCQ-12 domains are closely related, with all domains significantly contributing to the overall summary score (Table 2).

**Table 2:** Correlation among KCCQ -12 Domains scores and its summary score KCCQ: Kansas City Cardiomyopathy Questionnaire; r: Pearson coefficient, (degree of freedom =109), PL: Physical Limitation, SF: Symptoms Frequency, QL: quality of life, SL: Social Limitation.

		KCCQ12-PL	KCCQ12-SF	KCCQ12-QL	KCCQ12-SL	KCCQ12 Summary
KCCQ12-PL	r	1	0.450	0.345	0.492	0.758
	p-value	-	<0.001	<0.001	<0.001	<0.001
KCCQ12-SF	r	-	1	0.456	0.538	0.757
	p-value	-	-	<0.001	<0.001	<0.001
KCCQ12-QL	r	-	-	1	0.482	0.765
	p-value	-	-	-	<0.001	<0.001
KCCQ12-SL	r	-	-	-	1	0.811
	p-value	-	-	-	-	<0.001

Distribution of pharmacist interventions among HF patients

**Figure 2:**
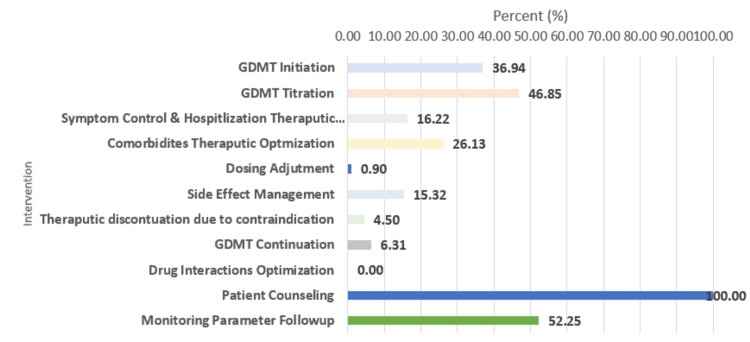
Distribution of pharmacist interventions among HF patients GDMT: Guideline-directed medical therapy

The distribution of pharmacist interventions among HF patients, as shown in Figure 2, highlights the most and least common activities. Patient counseling was the most frequent intervention, performed in 111 (100%) cases, emphasizing the importance placed on educating patients. Monitoring parameter follow-up (e.g., renal, hepatic, A1c, electrolyte, and lipid panels) was performed in 57 (52.25%) participants, indicating significant attention to ongoing assessment of the patient’s health indicators. GDMT titration and Initiation were also common interventions, conducted in 52 (46.85%) and 41 (36.94%) cases, respectively, suggesting a focus on optimizing GDMT for HF management. Other interventions, such as comorbidities therapeutic optimization (e.g., optimize diabetes, hypertension, and dyslipidemia therapeutics) (n=29, 26.13%) and symptom control and hospitalization therapeutics (e.g., patient education of diuretic dose adjustment at home) (n=18, 16.22%), were less frequent but still notable. Discontinuation of HF medication due to contraindication (e.g., stopping of ivabradine due to developed atrial fibrillation) or due to electrolyte disturbance (e.g., temporarily holding of mineral corticosteroid antagonist due to hyperkalemia or starting potassium binder to continue maximum and target doses of GDMT was reported in five (4.5%) participants. Dosing adjustment (n=10, 0.9%) and drug interactions optimization (0.0%) were rarely addressed, indicating fewer issues in these areas or a lower priority. Overall, this distribution demonstrates a pharmacist-driven approach prioritizing patient education, monitoring, and medication optimization for HF management (Figure 2).

Association between KCCQ-12 score levels and participants' social characteristics, comorbidities, and the received intervention(s) per patient

Table 3 shows the association between KCCQ-12 score levels and participants' social characteristics, comorbidities, and interventions received. Marital status was significantly associated with KCCQ scores (p=0.02), with married participants predominantly in the "Good to Excellent" category (89.4%), while divorced participants had higher representation in the "Fair to Good" category. Education level also showed a significant association (p=0.046), with participants having a university education more likely to report higher KCCQ scores. Those with fewer interventions generally reported lower KCCQ scores, while participants with three to five interventions were more evenly distributed across score levels. Readmission status was notably associated with lower KCCQ scores, with 37% of "Fair to Good" participants and 27.3% of "Good to Excellent" participants experiencing readmissions (p=0.07). DLP was another significant factor (p=0.029), with the absence of DLP more common in participants reporting better KCCQ scores. Other factors, such as diabetes (DM), hypertension (HTN), thyroid disorders, smoking status, and age, showed some differences across KCCQ levels but did not reach statistical significance. Overall, marital status, education, DLP, and readmission were key factors associated with higher KCCQ-12 scores, indicating a better QOL among participants in these categories.

**Table 3:** Association between KCCQ-12 score levels and participants' social characteristics, Comorbidities, and the received intervention(s) per patient KCCQ: Kansas City Cardiomyopathy Questionnaire; degree of freedom=1, DM: Diabetes mellitus, HTN: hypertension, DLP: dyslipidemia

			Poor to Fair (N=17)	Fair to Good (N=27)	Good to Excellent (N=66)	p-value	Chi-square	Effect size (Cramer's V)
Marital Status	Divorced	N	0	3	0	0.02	11.7	0.2
		%	0.0%	11.1%	0.0%			
	Married	N	17	21	59			
		%	100.0%	77.8%	89.4%			
	Single	N	0	3	7			
		%	0.0%	11.1%	10.6%			
Education Level	Elementary School Level	N	1	5	2	0.046	15.7	0.3
		%	5.9%	18.5%	3.0%			
	High School Level	N	3	8	16			
		%	17.6%	29.6%	24.2%			
	Higher Education Level	N	0	1	3			
		%	0.0%	3.7%	4.5%			
	Middle School Level	N	4	3	3			
		%	23.5%	11.1%	4.5%			
	University Level	N	9	10	42			
		%	52.9%	37.0%	63.6%			
Number of Interventions	No Intervention	N	3	2	3	0.86	8.5	0.2
		%	17.6%	7.4%	4.5%			
	One	N	1	2	2			
		%	5.9%	7.4%	3.0%			
	Two	N	4	7	17			
		%	23.5%	25.9%	25.8%			
	Three	N	5	9	16			
		%	29.4%	33.3%	24.2%			
	Four	N	2	5	16			
		%	11.8%	18.5%	24.2%			
	Five	N	2	2	9			
		%	11.8%	7.4%	13.6%			
	Six	N	0	0	2			
		%	0.0%	0.0%	3.0%			
	Seven	N	0	0	1			
		%	0.0%	0.0%	1.5%			
Readmission	No	N	16	17	48	0.07	5.3	0.2
		%	94.1%	63.0%	72.7%			
	Yes	N	1	10	18			
		%	5.9%	37.0%	27.3%			
DM	No	N	3	13	28	0.11	4.4	0.2
		%	17.6%	48.1%	42.4%			
	Yes	N	14	14	38			
		%	82.4%	51.9%	57.6%			
DLP	No	N	5	16	43	0.029	7.1	0.3
		%	29.4%	59.3%	65.2%			
	Yes	N	12	11	23			
		%	70.6%	40.7%	34.8%			
HTN	No	N	7	13	21	0.130	2.3	0.1
		%	41.2%	48.1%	31.8%			
	Yes	N	10	14	45			
		%	58.8%	51.9%	68.2%			
Thyroidism	No	N	17	24	61	0.34	1.9	0.1
		%	100.0%	88.9%	92.4%			
	Yes	N	0	3	5			
		%	0.0%	11.1%	7.6%			
Smoking	0	N	12	18	29	0.18	6.3	0.2
		%	70.6%	66.7%	43.9%			
	1	N	3	6	24			
		%	17.6%	22.2%	36.4%			
	2	N	2	3	13			
		%	11.8%	11.1%	19.7%			
Age groups(years)	<40	N	3	1	12	0.43	3.8	0.1
		%	17.6%	3.7%	18.2%			
	40-60	N	8	17	36			
		%	47.1%	63.0%	54.5%			
	>60	N	6	9	18			
		%	35.3%	33.3%	27.3%			

**Figure 3:**
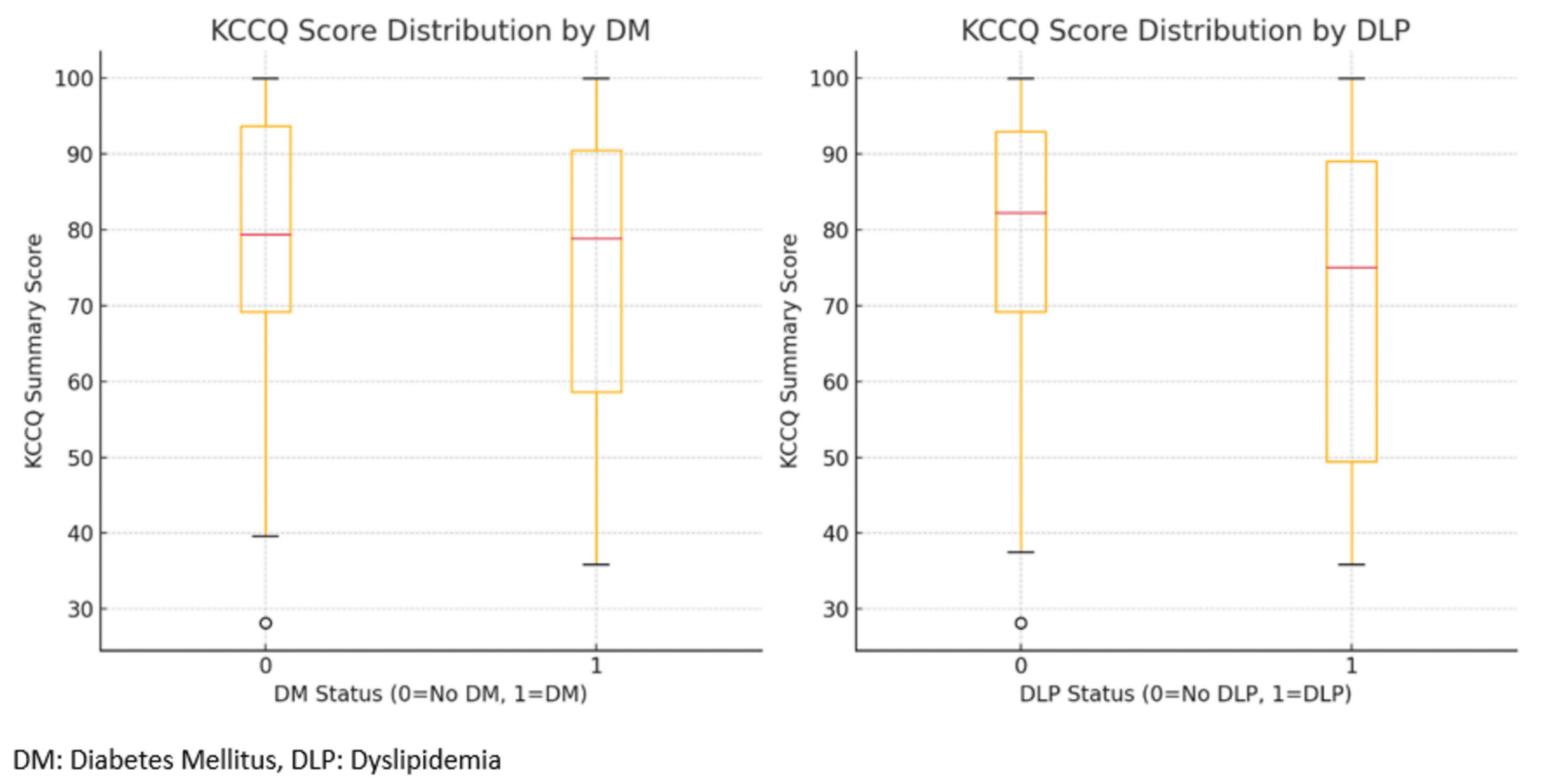
Box plot for KCCQ-12 summary score distribution by DM and DLP status KCCQ: Kansas City Cardiomyopathy Questionnaire; DM: diabetes mellitus, DLP: dyslipidemia

Figure 3 reveals that there is a notable variation in the KCCQ summary score distribution between individuals with and without DM and dyslipidemia (DLP). While the median scores appear relatively similar across DM status, there is a more noticeable drop in scores for individuals with DLP, suggesting that DLP may be associated with lower KCCQ scores, potentially impacting patients' QOL more than DM in this sample.

Table 4 shows a weak positive correlation (rho=0.183) between the KCCQ-12 summary score and the total number of interventions received by patients, with a p-value of 0.056. This suggests a trend where an increased number of interventions may be associated with a slightly higher QOL as reflected in the KCCQ-12 scores, although this correlation is not statistically significant (p>0.05).

**Table 4:** Correlation between KCCQ-12 Summary score and the total number of interventions received by patients ^a^ Spearman correlation (Degree of freedom =109)

		KCCQ12 Summary Score	Total Number of Interventions
KCCQ12 Summary	Rho^a^	1.000	0.183
	P value	-	0.056
Total Number of Interventions	rho	0.183	1.000
	P value	0.056	-

## Discussion

HF patients have a high mortality rate, and treatment aims to prolong survival, enhance quality of life, and prevent symptom progression. Poor health-related QOL is a significant concern for patients [28]. The KCCQ-12 test, which provides quick and consistent information, is linked to significant clinical outcomes in a diverse range of HF patients worldwide [29]. The adjusted mean KCCQ-12-SS (summary score) was found to be 20 and 10 points lower in Africa and Eastern Europe than in Western Europe, South Asia, and the Middle East [30]. The current study aimed to determine if HF clinics led by clinical pharmacists who specialized in cardiologic pharmacotherapeutic interventions may indeed enhance the QOL for patients with HF.

According to the results of the current study, in 60.4% of the valid responses, QOL was assessed to be Good to Excellent, showing that most patients have a good perception of their health. This is a good finding since it shows how well the existing interventions and treatment plans work to improve the patients' QOL. Still, a significant proportion of patients had lower health-related QOL, as seen by the 39.6% of responders who rated their QOL as Poor to Fair or Fair to Good. This emphasizes how important it is to keep looking for and fixing those problems causing these lower ratings. A prior cross-sectional correlational study that evaluated the QOL of HF patients in Saudi Arabia found that the patients' scores were low across all domains [4].

The results from earlier research employing the KCCQ have varied based on the setting and patient population. For example, the KCCQ is a valid and trustworthy indicator of health status, according to a systematic study analyzing its efficacy in assessing QOL among HF patients undergoing exercise training rehabilitation [31]. The review noted that KCCQ ratings improved for rehabilitation patients, indicating an improved QOL. Patients in New York Heart Association (NYHA) class I had significantly higher KCCQ scores than those in NYHA classes II and III, indicating improved health-related QOL, according to another study evaluating the QOL status in hypertrophic cardiomyopathy [32]. This implies that the intensity of their HF symptoms may greatly impact a patient's subjective QOL.

According to the study, the majority of HF patients received more than one intervention, which suggests that comprehensive care is urgently required. About 27% of the participants had three interventions, whereas the majority received two to four. Most responders, who might be newly diagnosed patients or those who are managing their condition in another way, did not receive any interventions. According to earlier research, pharmacist-led interventions can greatly enhance HF patients' outcomes, including diabetes, hypertension, dyslipidemia, quitting smoking, and hospitalization rates [33]. The percentage of patients who received guideline-directed drug therapy and the time to diagnosis were increased by a multidisciplinary strategy involving pharmacists, nurses, and other medical specialists [34].

According to the study, marital status significantly influences KCCQ scores, which measure quality of life. Married people are more likely to report higher QOL scores than divorced or single people. This implies that a partner's social or emotional support has a beneficial impact on the patient's perceptions of their quality of life. The study's findings, which show that marital status significantly impacts quality of life, align with earlier research. Married people often live longer and are in better health than single, divorced, or widowed people [35]. The notion that social support from a partner might have a good impact on perceived QOL was supported by a study conducted on patients with chronic renal diseases, which indicated that having a permanent partner was linked to improved mental QOL scores [36].

According to the KCCQ summary scores, health outcomes and educational attainment correlate significantly. Higher levels of education are frequently linked to improved health outcomes and health perspectives. Higher levels of education have been associated in a previous study with reduced mortality rates and better self-reported health status among HF patients [37]. The results imply that education may be a key factor in promoting better health outcomes by increasing health literacy, expanding access to healthcare resources, and encouraging healthier lifestyle choices.

According to the study, there may be an association between readmission history and KCCQ scores, with individuals without readmission history typically having higher scores. However, the Pearson chi-square test is near but falls short of the significance criteria. This implies that readmission history might impact KCCQ scores, although there isn't enough data to support this theory. Because the results are borderline significant, they should be regarded with caution. Previous studies that showed links between readmission history and patient-reported outcomes have demonstrated similar findings. Enhancing self-care after discharge improved KCCQ scores in another study [38,39].

DLP and lower KCCQ scores were significantly correlated in the study, indicating that those with DLP have a lower QOL. This is in line with earlier studies showing how DLP affects health outcomes [40,41]. Additionally, the study revealed that those without DLP scored higher on the KCCQ, indicating that better health and a higher QOL can result from efficient lipid control. A higher risk of CVD, which can have a detrimental effect on general health and quality of life, is linked to dyslipidemia. A healthy lifestyle, including diet and exercise, has also been linked to lower rates of CVD and all-cause mortality in individuals with dyslipidemia, according to earlier research [41].

The study found that whereas smoking and hypertension had a significant positive association with KCCQ scores, patients with DLP showed a significant negative correlation, indicating a lower quality of life. According to previous studies, people with DLP frequently have lower health outcomes and QOL because they are more likely to develop cardiovascular events and problems [42,43]. It's important to note that there is a positive correlation between hypertension and KCCQ scores, indicating that individuals with hypertension had higher perceived health status. This contrasts with other research, which generally shows that the burden of controlling hypertension and its risks has a detrimental effect on QOL [44,45]. Smokers' self-perception biases or other confounding variables could cause the study's positive association. The sample's KCCQ scores were not impacted independently by different factors such as age, diabetes mellitus, thyroid conditions, readmission history, or the total number of interventions. The results offer an in-depth overview of the variables affecting KCCQ scores in patients with HF. Although the detrimental effects of DLP are consistent with previous studies, the positive associations shown for smoking and hypertension warrant more research.

The current study showed a statistically significant association between HF patients' KCCQ-12 scores and the frequency of clinical pharmacist interventions. This implies that among patients with HF, a higher number of pharmacist interventions are linked to improved reported health status. According to a previous study, customized self-care interventions significantly raised KCCQ-12 scores. The study showed that treatments that addressed QOL and symptom management improved health status [39]. A systematic analysis that emphasized the role of pharmacists in optimizing HF drugs and enhancing patient outcomes came to similar conclusions [46]. According to a prior study, pharmacist-led interventions enhance medication adherence and may have a favorable effect on HF patients' outcomes [47]. In addition to highlighting the advantages of a multidisciplinary approach, the present study examined the efficacy of pharmacist interventions in managing cardiovascular disease (CVD), including HF.

Limitations of the study

The cross-sectional study design may limit the potential to demonstrate causality, and self-reported data could introduce bias into the study's findings on clinical pharmacist interventions for patients with HF. Incomplete data may also impact the validity and reliability of the results. Multiple interventions, uncontrolled variables, and a single-center study are confounding factors that may limit generalizability to different locations or practices. Borderline significance and interventional variability should be taken into account in statistical analysis because they may have an impact on the findings. Intervention standardization is vital for reliable comparisons and to guarantee that the results are generalizable to a larger population.

## Conclusions

According to the KCCQ, this study demonstrates the significant influence of clinical pharmacist interventions on the QOL of patients with HF. The results show that the number of interventions and better KCCQ scores correlate positively, indicating that comprehensive and customized pharmacist-led interventions can improve patient outcomes. Significantly, lower KCCQ scores were linked to dyslipidemia, highlighting the necessity of efficient lipid control to enhance perceived health status. Furthermore, smoking and hypertension revealed unexpected positive correlations with KCCQ scores, indicating the need for more research to fully comprehend these associations. Overall, the study contributes to the growing body of evidence demonstrating the value of clinical pharmacists in enhancing the QOL of HF patients.
